# Determination of roughness coefficient in 3D digital representations of rocks

**DOI:** 10.1038/s41598-022-15030-y

**Published:** 2022-06-25

**Authors:** Leonardo Scalco, Leandro Tonietto, Raquel Quadros Velloso, Graciela Racolte, Luiz Gonzaga, Mauricio Roberto Veronez

**Affiliations:** 1 Vizlab-X-Reality and Geoinformatics Lab, Unisinos University, São Leopoldo, 93.022-750 Brazil; 2 Post Graduate Program in Applied Computing, Unisinos University, São Leopoldo, 93.022-750 Brazil; 3 Department of Civil Engineering, PUC University, Rio de Janeiro, 22.451-900 Brazil

**Keywords:** Computer science, Statistics, Civil engineering

## Abstract

The roughness property of rocks is significant in engineering studies due to their mechanical and hydraulic performance and the possibility of quantifying flow velocity and predicting the performance of wells and rock mass structures. However, the study of roughness in rocks is usually carried out through 2D linear measurements (through mechanical profilometer equipment), obtaining a coefficient that may not represent the entire rock surface. Thus, based on the hypothesis that it is possible to quantify the roughness coefficient in rock plugs reconstructed three-dimensionally by the computer vision technique, this research aims to an alternative method to determine the roughness coefficient in rock plugs. The point cloud generated from the 3D model of the photogrammetry process was used to measure the distance between each point and a calculated fit plane over the entire rock surface. The roughness was quantified using roughness parameters ($R_a$) calculated in hierarchically organized regions. In this hierarchical division, the greater the quantity of division analyzed, the greater the detail of the roughness. The main results show that obtaining the roughness coefficient over the entire surface of the three-dimensional model has peculiarities that would not be observed in the two-dimensional reading. From the 2D measurements, mean roughness values ($R_a$) of $0.35\,\upmu \hbox {m}$ and $0.235\,\upmu \hbox {m}$ were obtained for samples 1 and 2, respectively. By the same method, the results of the $R_a$ coefficient applied three-dimensionally over the entire rocky surface were at most $0.165\,\upmu \hbox {m}$ and $0.166\,\upmu \hbox {m}$, respectively, showing the difference in values along the surface and the importance of this approach.

## Introduction

In studies related to geomechanics, knowing the basic properties of rocks, such as their mechanical resistance, hydraulic flow, and fluid percolation, is essential to understanding their behavior in critical situations^1,2^. In this sense, the areas that present these properties are the fractures (joints), defined by the relationship between two rock surfaces separated at some points but in contact at others^2,3^. The anisotropic morphology of natural rock mass joints constantly affects their shear behavior^4,5^.

Some surface properties of these fractures are significant for their mechanical, hydraulic, thermal, and transport behavior, and the fluid flow in these places is superior to in the rock matrix^6^. One of these properties is the roughness, which is crucial in the stability of rock masses, and essential in geotechnical studies or engineering works^5,7–10^.

The roughness study is applied in the risk assessment for possible water percolation in tunnels and natural coal mines, identification, and exploration of geothermal reservoirs, the capture of $CO_2$, studies for disposal of underground deposits of radioactive material, capture and use of groundwater, and classification and study of natural well fractures as a means of disposal and storage of crude oil^7,11–13^. To determine the roughness in rock masses joints, research was carried out to establish a relationship between the joint opening distribution and the fluid conductivity, and a greater roughness on any surface hinders the flow and facilitates the union between separate layers^9,14^.

The method most used to quantify the fracture roughness is the joint roughness coefficient (*JRC*)^15^. It is conceptualized as the relation between the standard deviation of the variable opening and the mechanical opening and is obtained by comparing the fracture geometry and the standard profiles using a profile scale^15^. Barton et al.^15^ proposed a values classification of *JRC* from a sample of 136 natural rocks, defining a scale to divide the roughness coefficients from 0 to 20 into groups. The rock’s roughness coefficients are obtained through the visual comparison of two-dimensional profiles obtained in the field and these tabulated values. Despite the importance of this research, the method has limitations like the visual comparison, and the results are influenced by the professional’s experience^9,16^. Furthermore, define only two-dimensional (2D) linear profiles, not covering the entire surface of the rock^6^.

In this scenario, the use of digital representations in the materials properties characterization is increasing due to the precision of the profile obtained and the facility to replicate a sample to reach a larger number of tests^16^. A reliable 3D digital representation is obtained from two-dimensional images (photogrammetry) or through a dense point cloud containing positioning information generated by laser scanning^17^. Through photogrammetry, the data acquisition has a higher resolution, and with image processing software, it is possible to reconstruct three-dimensional models^8,17^.

These techniques have advantages over conventional acquisition methods of physical samples of rock, such as the obtainment in inaccessible places, less dependence on the professional working in the field experience, the possibility of a greater quantity of rock samples with less manual work, and the possibility of 3D representation of the entire rock sample^16,18^. Another incentive for the increment of 3D models is the improvement of computational methods. Currently, two-dimensional images and referenced points obtained by photogrammetry or by laser scanning are efficiently processed by algorithms, reducing manual work and generating more reliable results^17^.

Some strategies to measure the roughness coefficient in rock mass joints using 3D models were developed to support the traditional techniques. Therefore, it is natural to carry out research that applies functional methods but is used in other areas of study to obtain roughness coefficients in digital representations of rocks^16,17,19^. Tonietto et al.^20,21^ calculates the roughness of a block substrate (used to build masonry walls) in height and area measurements to favor adhesion by contact area. The authors used a point cloud obtained by laser scanning (LiDAR), where each point contains position and elevation information. The roughness of the block was measured between the height difference of each point and a created plane that best represented the group of points (fit plane), forming peak areas (group of points above the fit plane) and valley areas (group of points below the fit plane)^21^.

Through the related works, it is possible to find limitations and research gaps to be filled. Some methods used two-dimensional linear results, which do not represent the entire rock surface^17^. The use of laser scanners in their methodology is another limitation found. This equipment has a high cost of acquisition and operation, and is laborious, as the rock samples require greater preparation than photogrammetry. With photogrammetry, it is possible to acquire rock samples by the non-destructive method in the field. In addition, it generates higher productivity and lower cost compared to laser scanners^22^.

The 3D roughness measurements on rock surfaces are essential for obtaining this coefficient to be realistic on the entire rock surface. In this sense, the method for results analysis in hierarchical division allows obtaining the average roughness over the total surface of rocks or rocky outcrops^20^. The main advance of this method in relation to the studies shown above is the ease of increasing (or decreasing) the level of details of results according to the interest of the work performed, changing the analysis division. Furthermore, it can be applied both at the laboratory rock sample level and for application on large rocky outcrops.

This work aims at an alternative method to obtain the roughness coefficient in rock plugs samples. This objective seeks to confirm the hypothesis that it is possible to quantify the roughness coefficient in 3D reconstructed rock plugs using the computer vision technique. However, it was necessary to make adaptations to the method^20^. The achievement of results was in 2D linear measures (in addition to the three-dimensional), so it can be directly validated and compared. Another adaptation performed to the method to geomechanics was obtaining the roughness parameters for the global plane of the rock sample and the representation of the roughness data in a localized way (node grid of the quadtree)^20^.

## Roughness identification in the hierarchical structure

The method^21^ quantifies the surface roughness of blocks through a roughness visual signature, calculated from parameters of hierarchically organized regions. The point clouds of the ceramic block samples were generated by laser scanning. The method developed analyzes point cloud data and roughness parameters through different scales in a hierarchical structure. In this scenario, roughness parameters are calculated for all points at all levels of spatial division. In this way, the surface roughness is measured globally (highest level of the structure—no division) and locally (lowest level—three divisions), totaling 64 squares for individual analysis of average roughness. In the work of Tonietto et al.^20^, three division levels were used to compute localized roughness (with more details), but the method allows subdividing into more levels.

**Figure 1:**
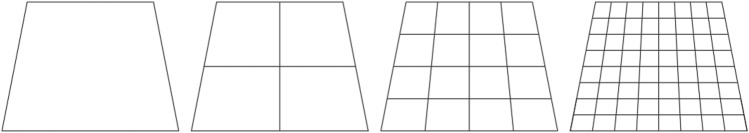
Example of hierarchical structure division (quadtree root, first subdivision, second subdivision and third subdivision, respectively).

The organization and division of the hierarchical structure follow a model known as a quadtree, wherein division behavior always has the same procedure (division into four equal parts—Fig. 1), regardless of the size of the analyzed sample. In this type of structure, the original quadrant has similar information to the mean established for the four original quadrants from its division. Thus, the subdivisions needed for each sample will depend on the user who will perform the test. As a result of this division model, each square resulting from the subdivision will have an average roughness result. Thus, the greater the degree of division, the smaller the squares generated and the more detailed the average roughness will be^21^.

For this method, the authors^20^ defined roughness as the average height of the point in relation to the fit plane. This fit plane was calculated considering the orientation and slope of the surface from the least-squares product technique, which makes a regression of the three-dimensional data to a plane in order to represent the fit plane in the entire set of points. The distance between the points and this fit plane is the roughness at that location. The points below this plane generate valley points, and the points above this reference generate peak points^21^.

Thus, the roughness parameters from the plane coefficients were calculated, defining the mean roughness ($R_a$). A local analysis of the results is essential for determining a roughness signature composed of the values of each region subdivided in a hierarchical way. In this division, the values found in the last level are the minimum area values defined by the user for roughness evaluation. With this roughness signature ($R_a$), the authors compared each surface in a standard way. After roughness identification, the results are processed to generate graphs and other parameter information. In this stage, the mean roughness coefficients ($R_a$) are obtained^20^.

## Method

**Figure 2:**
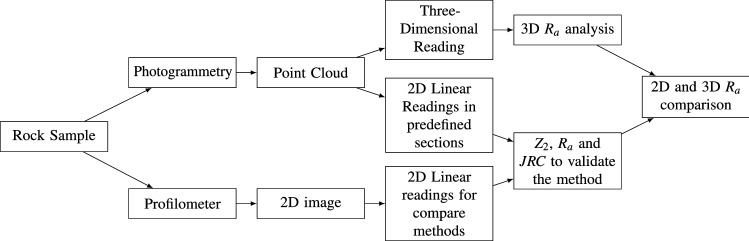
Methodology flowchart.

The proposed method for determining the roughness coefficient in rocks from three-dimensional digital models (Fig. 2) consists of acquiring the cloud of points from the surface of the rock sample by employing digital photogrammetric and 3D readings for the calculation of the $R_a$ and *JRC*. Subsequently, the $Z_2$ and *JRC* are calculated from the 2D analysis method to compare and validate the methodology.

### Acquisition and preparation of rock samples

**Figure 3:**
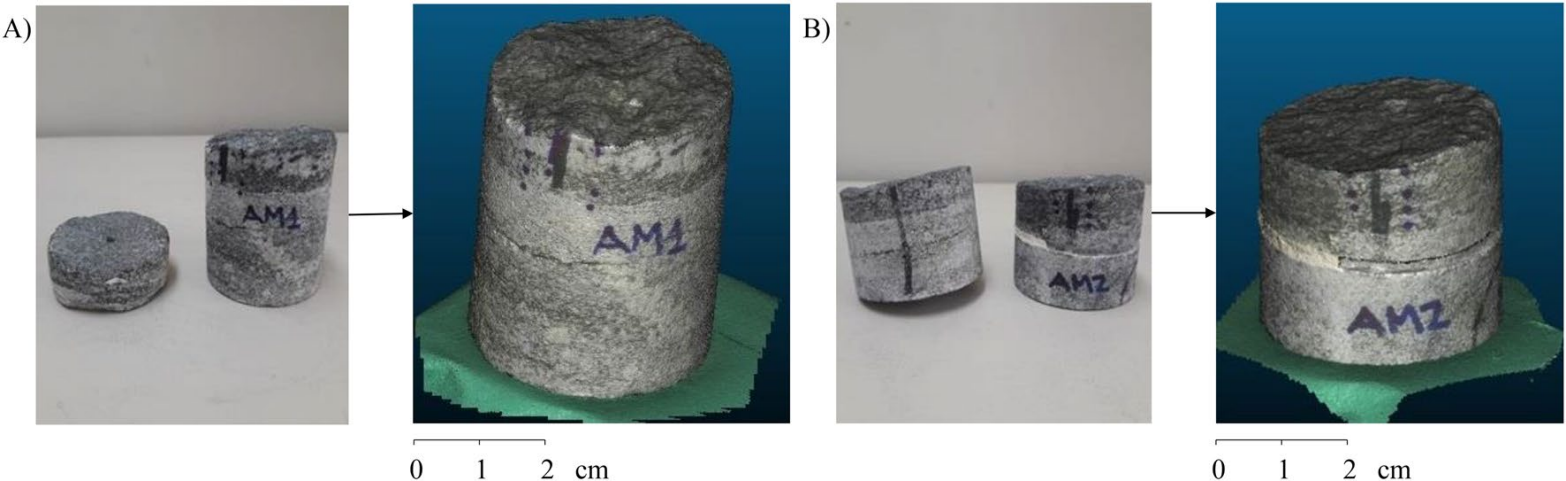
Used samples and models from photogrammetry: (**A**) Sample 1 and (**B**) Sample 2.

The rock samples used in the research are from the Bangú quarry in the municipality of Rio de Janeiro, Brazil (Fig. 3). This place is predominantly composed of gneiss rocks. The roughness analysis in them is capital for the identification, classification, and mechanisms of fracture formation. Gneiss rocks are metamorphic rocks. They originated from pre-existing rocks through chemical, mineralogical, textural, structural changes, or a combination of these factors, and this transformation occurs through changes in temperature and pressure^23^. Thus, the roughness and fluid flow characteristics in metamorphic rocks are related to the rocks that form them, requiring a particular study of each rock sample to understand their behavior^24^.

In the first stage of preparing the rock samples, a block is removed from its original location, taking care to identify the side parallel to the fracture. This identification is essential to define the experiment to generate the surface to obtain the roughness coefficient that reproduces the environment. For the methodology of this research, rock samples need to be in a format with smaller dimensions called a plug^23^. This plug is cylindrical in shape and obtained through equipment that penetrates the rock block and collects a cylindrical sample from that block. In the present study, plugs were cut with an average diameter of 5.3cm and height ranging from 8cm to 12cm. In this study, to obtain the rough surface of the rock, the direct shear test was performed. This test is carried out in the laboratory, causing a displacement effort of the rock sample.

### Photogrammetry point cloud generation

In this research, the point cloud was obtained by generating three-dimensional models of rock samples from photogrammetry. This method aims to create 3D models from two-dimensional images^25^. This technique has two stages in its process. The first is the acquisition of photos, and the second is processing in software.

For photos acquisition, it was employed a support table, a mini studio, a centimeter ruler to measure the sample size, and a Canon Digital Camera model EOS Rebel T6i. The mini-studio utilized to capture the photographs has only one open side and is composed of green walls, ceiling, and background due to the contrast with the photographed samples. In addition, it has a turntable with $360^\circ$ angular markings, so it is possible to rotate the entire rock without moving it. Also, there are three targets marked on the turntable to position and scale the rock sample. Another element of its composition is the LED lighting inside.

**Figure 4:**
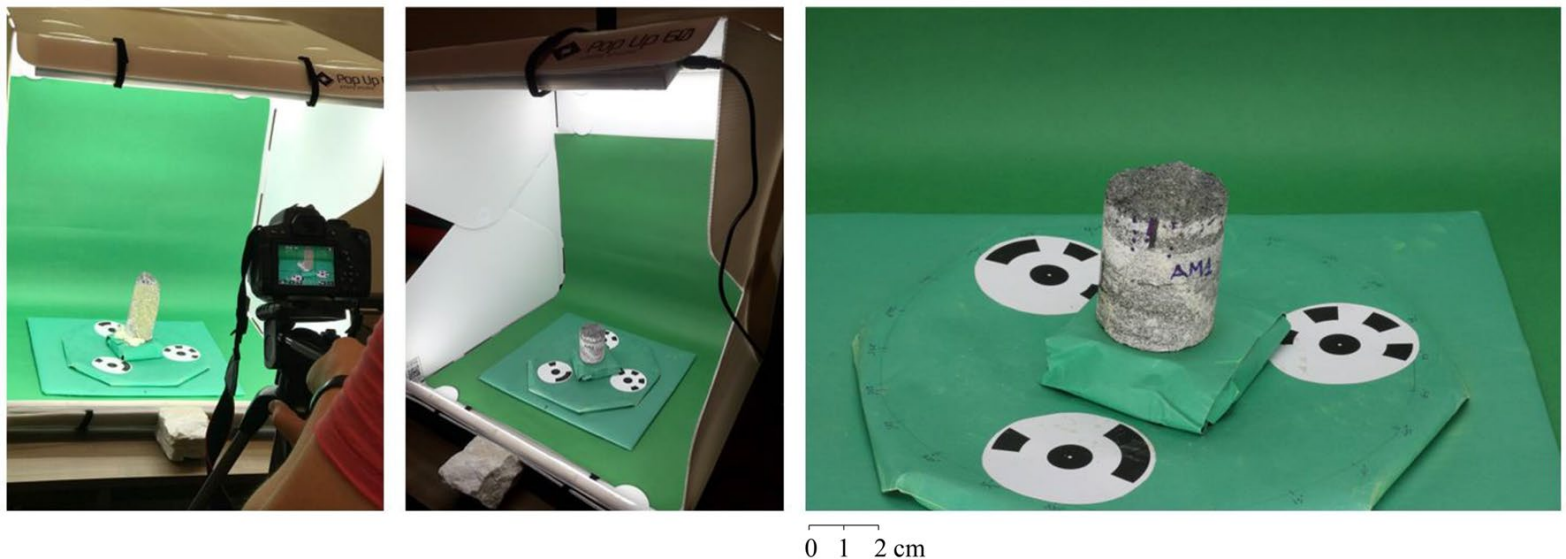
Photogrammetry process detailing all the elements as well as their positioning throughout the process.

The rock samples were photographed at different angles to search for a mapping that best represents the original sample. Thus, the rock sample was photographed along its entire perimeter every $15^\circ$, resulting in 24 photographs. The camera was at a distance of approximately 90cm from the rock sample and at an angle of $30^\circ$. The acquired images have 6000 pixels $\times$ 4000 pixels (width $\times$ height) of dimensions, a vertical and horizontal resolution of 72 dpi, 24-bit intensity, and representation of color in RGB (Red; Green; Blue), and a focal length of 50 mm. Figure 4 details the photogrammetry process performed for one of the samples. In the Figure, it is possible to observe all the previously detailed elements and their positioning throughout the process.

**Figure 5:**
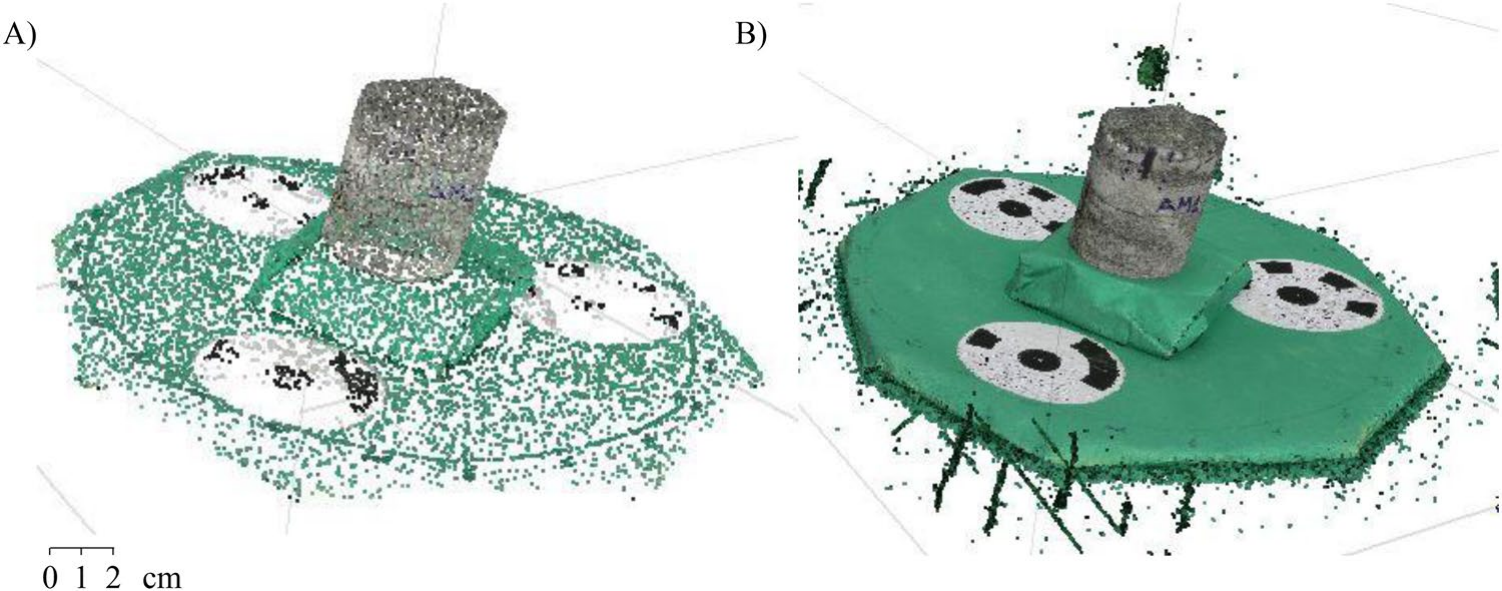
(**A**) Sparse and (**B**) dense point cloud$^1$.

After photo acquisition, the second part of the photogrammetric process consists of data processing in the 3D software Agisoft—Metashape (Agisoft Metashape 1.8.3—https://www.agisoft.com). In this stage, the photographed rock sample was imported separately for aligning the photos and optimizing this alignment, generating the sparse point cloud and the dense point cloud (Fig. 5). The sparse point cloud is to identify common points between the photos acquired in the first step, and the dense point cloud has greater detail and punctual precision. Subsequently, the alignment of the point cloud was to identify the position information of the points. From there, the mesh generation was performed by triangulation and texture generation.

**Figure 6:**
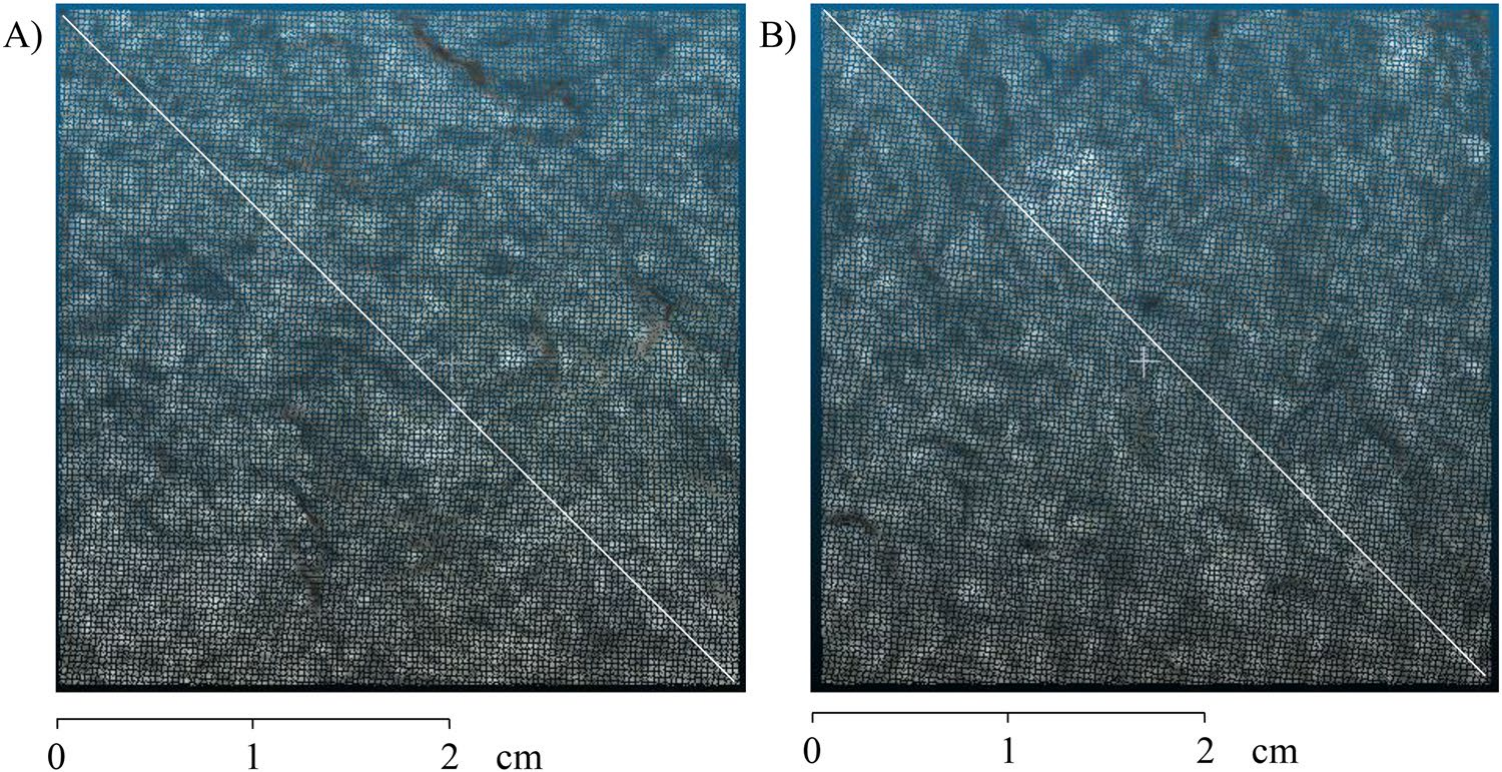
Square samples of 3.5 cm $\times$ 3.5 cm. The diagonal line represents the location of the two-dimensional readings: (**A**) Sample 1 and (**B**) Sample 2.

After the 3D model generation, the point cloud can be represented in a spatial format with 3D position coordinates of each point. The quality of roughness data in the rock sample is related to the distance between the points obtained through the model. The greater the density of the point cloud, the greater the precision and detail of the peak and valley areas. In this survey, the points are apart from each other on average by 0.1 mm (Fig. 6).

### Roughness coefficient by three-dimensional method

The method used to obtain the roughness coefficient considers a dense cloud of points with three-dimensional position coordinate information. As a first step in the methodology, the point clouds from each rock sample were cut to partially exclude the studio background and turntable base. This process was necessary to exclude outliers from the photogrammetric process. After, it is needed to cut the surface of each point cloud quadratically with dimensions of 3.5 cm $\times$ 3.5 cm. This process is relevant so that there is no interference from the edge of the plugs rock samples in the roughness calculation. In these places, the edges can be identified incorrectly as roughness. The surfaces of roughness analysis resulting from this process are in Fig. 6.

The software^20^ reads each of these points individually and automatically to obtain the necessary information for the calculation of $R_a$ (Mean Roughness—Eq. (1)^26^) and $Z_2$ (Root mean square deviation of the profile derivative—Eq. (2)^27^).

The average roughness ($R_a$) is the arithmetic mean of the absolute values, obtained by summation of the distance between the roughness measurement points to the fit line defined for the profile ($y_{i}$) and the total length sample (*L*)^26^.

1 $$\begin{aligned} R_a = \frac{1}{L}\sum _{0}^{L} |y_{i}| \end{aligned}$$

For calculating $Z_2$ for equal ranges, it is necessary to perform the sum of squares of the differences in adjacent coordinates (*y*) along the entire length of the sample (*L*). This sum is divided by the range (*x*), and applied to the square root^27^.

2 $$\begin{aligned} Z_2 = \frac{1}{L} \times {\sum \limits _{i=1}^{N-1}(\frac{(y_{i+1} - y_{i})^2}{x_{i+1} - x_{i}})^\frac{1}{2}} \end{aligned}$$

For calculating the Joint Roughness Coefficient (*JRC*), a correlation was developed with the parameter $Z_2$ through Eq. (3) for an interval of 1 mm^28^.

3 $$\begin{aligned} JRC=64.22(Z_2)-2.31 \end{aligned}$$

The software import the point’s coordinates through a text format file. In the imported file, each line contains information for each point in the cloud divided into three columns containing the three-dimensional positioning information (First column: *x* coordinate; Second column: *y* coordinate; and third column: *z* height coordinate). A code sequence in the same programming language identifies the coordinate of the points and the graphical results and fit planes displayed to the user. The generation of the fit planes occurs through the calculation of three individual vectors for each node of the quadtree. The hierarchical division of the rock sample surface calculated was 64 squares. This division is the result of 3 subdivisions performed to generate the quadtree.

In the last part of the method, the code loaded the points again to calculate the valley areas. In addition, in this part of the method, the graphic results are presented through a web application (valley areas, fit planes, unidirectional $R_a$ differences graph, and roughness coefficient dispersion histogram).

**Figure 7:**
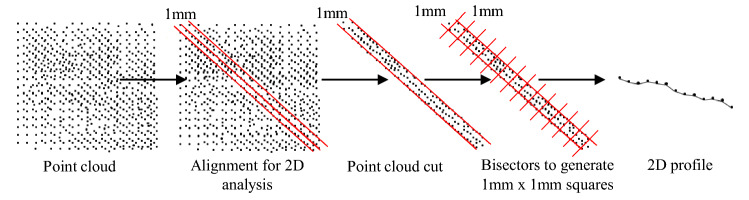
Method for 2D data acquisition.

For validating this present methodology, a comparison with a 2D method already used was made. In addition to the three-dimensional readings obtained over the entire surface of the rock sample, 2D linear results were also obtained at identical locations by the two methods. For this, the cloud of points was cut again, generating a line of points with a thickness of 1 mm. The rock samples were marked in the places of the first 2D results to be accurately measured. For this, was added to the code, a new linear read configuration. This new setup separates the point cloud by a thickness of 1 mm and calculates bisectors along the entire line of points, segregating the alignment into small squares of 1 mm $\times$ 1 mm. A mean solid line between the first and last point of the line of points is created to calculate the segments along the entire solid line with a distance of 1 mm from each other. Then the cloud points are plotted along the line. The maximum elevation point in each generated square is calculated to produce a linear profile. This process aims to create a profile that simulates the profilometer to compare the two methods. An example of the process is described in Fig. 7.

### Roughness coefficient by two-dimensional linear method

A method validates the methodology of roughness coefficient by hierarchical structure applied to geomechanics through the parameter $Z_2$ as a variable in the *JRC* calculation^29^. As an initial part of the process, the profile is printed on a profilometer, which serves to mold profiles and surface roughness designs for shear tests^15^.

It has a length of 15cm, a depth of 50 mm, and juxtaposed individual needles that change their height according to the surface in contact. To profile the 2D rock surface roughness, the profilometer is pressed against the rock sample surface. In the second part of the method, the created profile is positioned to cast a shadow on a white background and is produced a backlit photograph.

The following steps are computational, and it is necessary to make the images in monochrome format (black and white). That images are processed using MATLAB (MATLAB 9.1.0—https://www.mathworks.com/products/matlab.html) software, which reads and converts the image matrix to generate a *y* vector. The MATLAB (MATLAB 9.1.0—https://www.mathworks.com/products/matlab.html) algorithm calculates $Z_2$ (Root mean square deviation of the profile derivative—Eq. (2)^27^) and *JRC* (Joint roughness coefficient—Eq. (3)^28^).

The method validation applied ten profiles to draw corresponding vectors and assess their accuracy. The same methodology was used with vectors reconstructed from previous images, besides being visually compared.

### Comparison between the methods

For validating the methodology applied to geomechanics, the results found must have statistical importance. The objective is to determine if there is a significant difference (or advantage) between the two methods.

For this validation, the T-Student hypothesis test was used. The test is recommended for statistics with a sample population of less than 30 and when the sample subjects are divided into two groups (group A and group B)^30^. This estimate is used when the population standard deviation is unknown. It is important to note that statistics are basically about probabilities, as their result does not reflect an absolute value but a probability of an event occurring.

The research estimated the error rate and statistical power of the one-sample and two-sample T-tests for normally distributed populations. A paired T-test is feasible with an extremely small number of samples. It is concluded that there are no objections to using a T-test with *n* = 2. The derivation for the paired T-Student test is in the Eq. (4) (${\bar{d}}$ is the mean of the sum of the differences between the values of each sample and the average between them, $s^2(d)$ is the variance of the differences, and *n* is the sample number).

4 $$\begin{aligned} t = \frac{{\bar{d}}}{\sqrt{\frac{s^2(d)}{n}}} \end{aligned}$$

## Results

The results of this study are divided into three parts: results from a three-dimensional method^20^, results from a two-dimensional method^29^ and a comparison between the two methods.

### Results from a three-dimensional method

**Figure 8:**
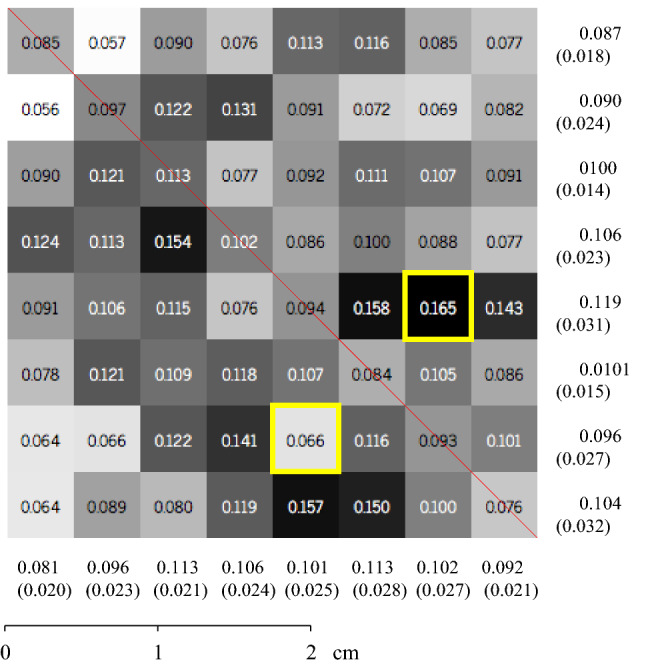
Average roughness for each subdivision square for Sample 1.

**Figure 9:**
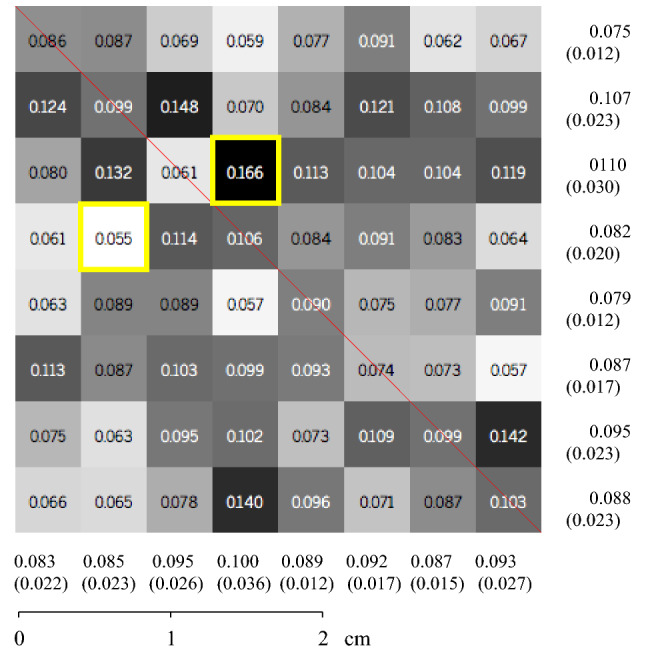
Average roughness for each subdivision square for Sample 2.

First, the roughness information in each subdivision performed in the hierarchical structure is shown (Figs. 8 and 9).The coloration of each quadtree represents how rough that place is. The color variation is represented from white scale (fewer roughness places) to black (roughness locations), ranging in grayscale to median values between maximum and minimum. It is important to note that it does not represent the depth of valleys and peaks. The red alignment is where the 2D linear results were also obtained. The squares in yellow highlighted the highest and lowest roughness in each rock sample.

**Figure 10:**
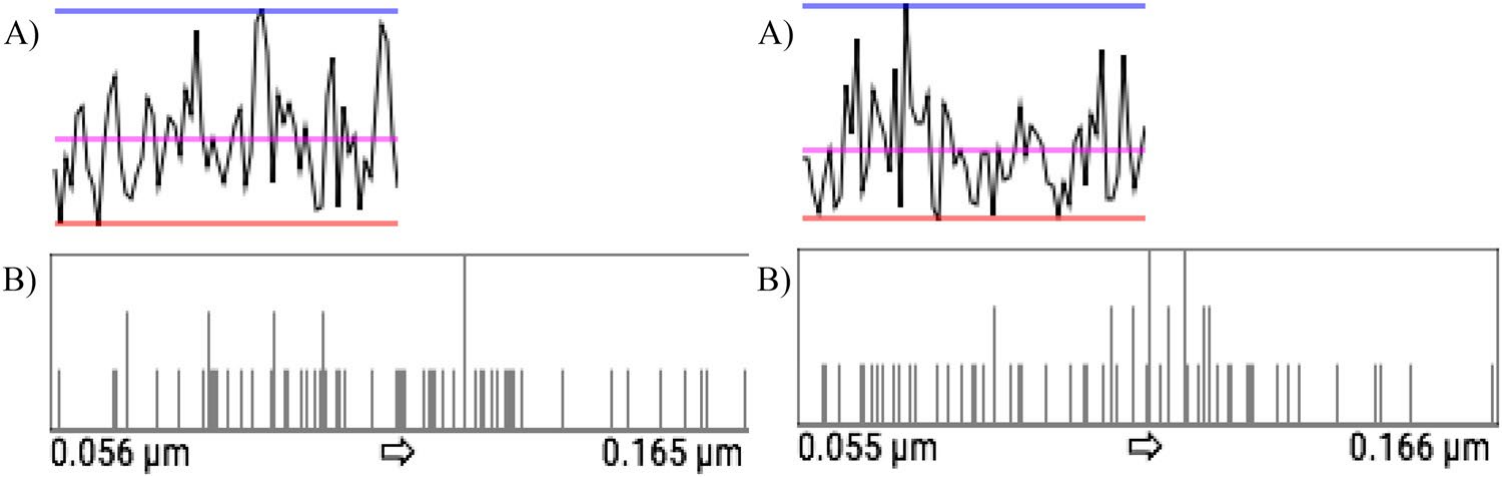
(**A**) Linear distribution of surface roughness values and (**B**) Histogram of roughness value distribution: Sample 1 and Sample 2, respectively.

Figure 10A shows the maximum (blue line), minimum (red line) roughness, and the average—the sum of the average roughness of each quadtree ($R_a$), divided by the number of quadtrees (magenta line). The roughness distribution in each square is also presented linearly. In Fig. 10B it is possible to see the histogram of roughness value distribution along each sample.

**Figure 11:**
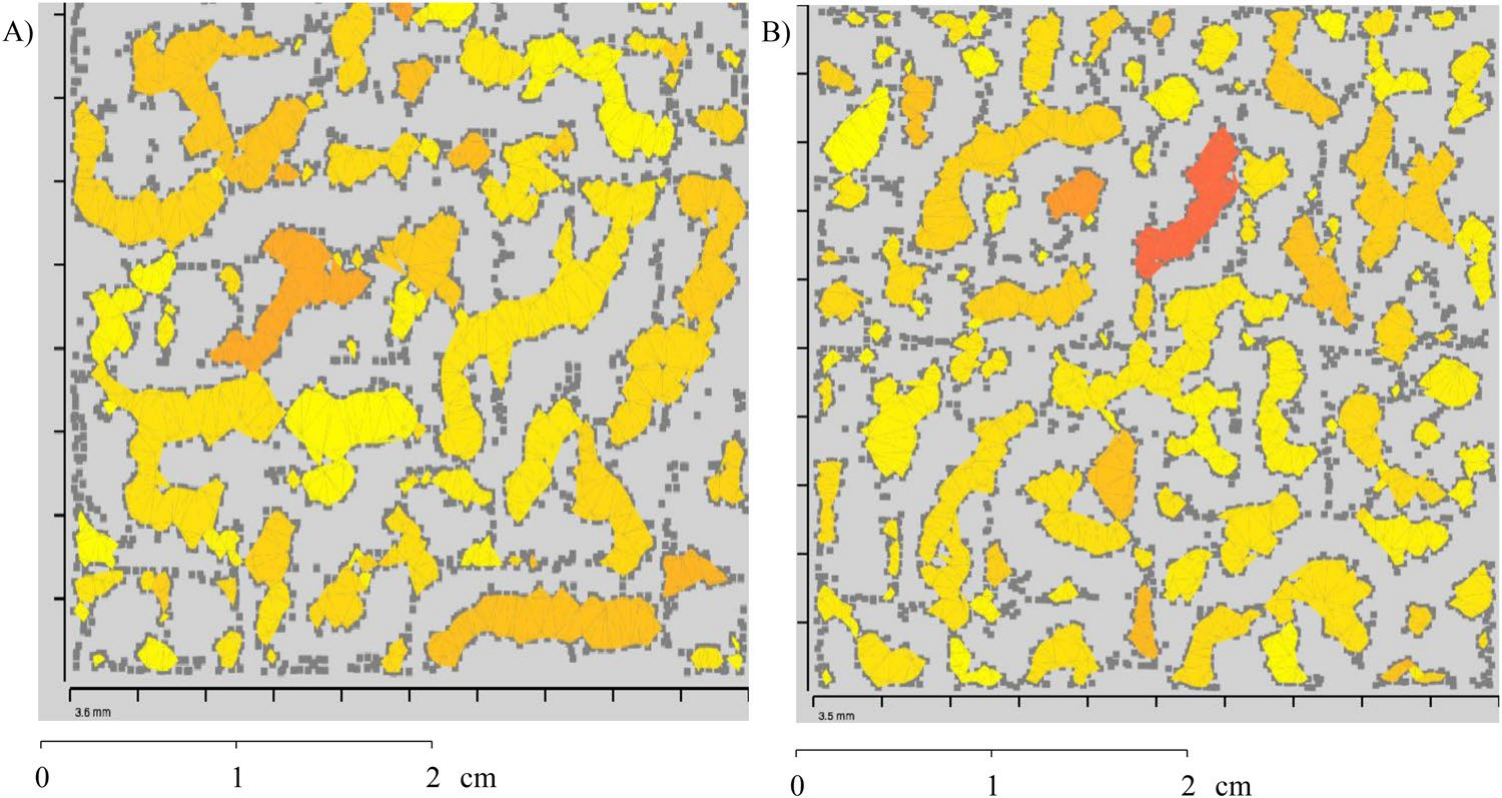
Valley areas and peak areas on the sample surface: (**A**) Sample 1 and (**B**) Sample 2.

Another result using this methodology shows the points where valley areas occur (yellow areas in Fig. 11—the darker the color, the deeper the area). The color is defined by evaluating the highest valley height in the area. These data can be used for fluid flow prediction studies in rock masses and to evaluate the mechanical behavior of rocks.

To define the valley area index parameter ($\triangle {_T}$), the division between the surface valley area index ($\triangle {_S}$) and the total surface area ($\square {_{surface}}$) is performed using Eq. (5). The total surface area and the valley area are calculated through polygonal triangulation between the points connected with reference to the neighboring point. The first step is to separate the sample into regions where the points are below the adjustment level. Then, the software connects by means of triangulation the neighboring points that are below the fit plane, creating regions known as lakes. In this triangulation, only areas with edge points (places where the distance between the point and the fit plane is zero) and their inner points are used. After triangulation, it is possible to form polygons (lake boundaries), where the area is calculated through the dimension of the created triangles. This process makes the points have pixel behavior, where a binary image (black or white) is created informing if the point is above or below the fit plane. In this scenario, a valley region is a set of connected black pixels. The algorithm identifies all black points and observes the behavior of the neighboring pixel until all areas are established^21^.

5 $$\begin{aligned} \triangle {_T} = \frac{\triangle {_S}}{\square {_{surface}}} \end{aligned}$$

As general results found for Sample 1, it is possible to observe that the maximum average roughness ($R_a$) found corresponds to $0.165\,\upmu \hbox {m}$ and the minimum roughness found to $0.056\,\upmu \hbox {m}$. The valley area percentage corresponds to 33.46% of the total area. In Sample 2, the maximum and minimum roughness ($R_a$) were $0.166\,\upmu \hbox {m}$ and $0.055\,\upmu \hbox {m}$, respectively. The total area of the valley found was 31.15% of the total area analyzed.

Through the adaptations made to the method of hierarchical structures, were obtained the 2D results. These results are in pre-defined sections in the rock sample for later comparison between this method and the method used for its validation. For Sample 1, the average roughness found ($R_a$—Eq. (1)) was $0.35\,\upmu \hbox {m}$ and the coefficient $Z_2$ (Eq. (2)) obtained was 0.42 with a sampling interval of 1 mm. Through Eq. (3), the joint roughness coefficient (*JRC*) through the $Z_2$ was 22.86. With the mean roughness coefficient ($R_a$) as a variable, the result found for *JRC* was 19.18. For Sample 2, the average roughness ($R_a$—Eq. (1)) was $0.235\,\upmu \hbox {m}$ and the coefficient $Z_2$ was 0.284 with a sampling interval of 1 mm (Eq. (2)). The joint roughness coefficient (*JRC*—Eq. (3)) through the coefficient $Z_2$ was 15.565 and the *JRC* through the average roughness ($R_a$) was 12.75. In Table 1 there is a description of all the results obtained through our methodology.

**Table 1:** Our general methodology results.

Rock sample	Linear $Z_2$/*JRC* results	Average 2D linear $R_a$/*JRC* results)	Average 3D $R_a$ results
1	0.42/22.86	0.35/19.18	0.100
2	0.284/15.565	0.235/12.75	0.090

### Results from a two-dimensional method

**Figure 12:**
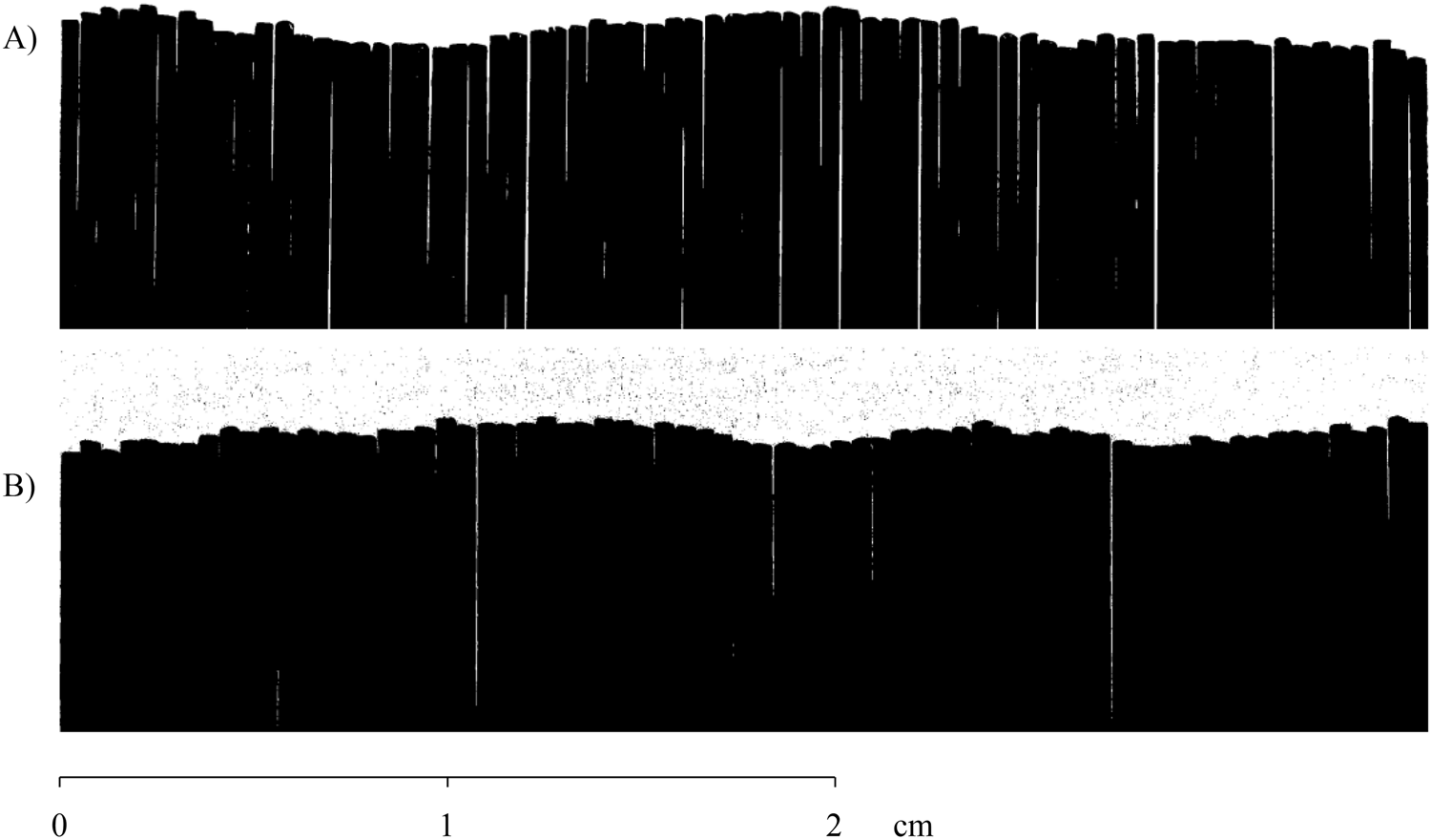
Linear two-dimensional results: (**A**) Sample 1 and (**B**) Sample 2.

The results of joint roughness coefficients (*JRC*) by 2D linear metrics^29^ were obtained for each sample (Fig. 12). For Sample 1, the parameter $Z_2$, also calculated using Eq. (2), has a value of 0.5440 and a *JRC* of 28.973 (Eq. (3)). For Sample 2, the value of $Z_2$ found was 0.3331 and *JRC* = 18.293.

The methodology used to obtain these results was based on two-dimensional linear readings performed using a profilometer and image processing in the MATLAB (MATLAB 9.1.0—https://www.mathworks.com/products/matlab.html) software. These results were obtained in the same alignment (Figs. 8 and 9) previously defined to compare the two methods and validate the methodology proposed in this work.

### Comparison between the two methods

For comparing the 2D linear results of the two methods, was used the T-Student test. This test was used due to the restriction of two in the number of rock samples used in this study (recommended for sample populations smaller than 30)^30^. The paired T-Student test (Eq. (4)) aims to compare means for the same group of subjects with a variable, with groups of *n* = 2 samples. For the comparison, the results of the coefficient $Z_2$ were used (Table 2).

**Table 2:** $Z_2$/*JRC* linear results for 2D comparison and validation of our methodology.

Rock sample	$Z_2$/*JRC* (our methodology)	$Z_2$/*JRC* (comparative methodology)
1	0.42/22.86	0.544/28.973
2	0.2837/15.565	0.3331/18.293

For Sample 1, we obtained a mean value of the differences ${\bar{d}}$ = 0.124, variance of the differences $s^2(d)$ = 0.003844 with the number of samples *n* = 2. A T-Student test value of 2.8284 was obtained. For the Sample 2, a mean value of differences ${\bar{d}}$ = 0.0491 was reached, variance of differences $s^2(d)$ = 0.000603 for the same number of samples *n* = 2. The value for the T-Student test was 2.8277. For a two-tailed degree-of-freedom Student’s t-test, the value of the tabulated area for acceptance of the null hypothesis is 12.706. As the value found was lower than the tabulated value, it is possible to accept the hypothesis that there is no significant difference between the results of the two rock samples and those of the comparative method.

## Discussion

In this section, the results are analyzed in order to prove (or refute) the hypothesis that the results obtained through three-dimensional reading are essential in geomechanics due to the non-regularity of the rock surface. The first part of this chapter, are discussed the 2D linear results using the two methods. Subsequently, the three-dimensional results of the entire rock surface are evaluated and compared with the two-dimensional linear results.

Through the results of both methods, it is possible to observe differences between the values obtained for the coefficients $Z_2$ and consequently in the *JRC*. Other research shows that the results for samples with intervals of 1 mm (criterion used in this study) have an average difference of 13.75% and 12% for a $R^2$ of 0.893 for the compared samples^17,22^. In our results, the difference in values for Sample 1 between the two methods was 21.1%, and for Sample 2 was 14.91%. It is important to note that the method used for comparison in this research^29^ also presents an average error close to that observed in the literature. Therefore, for more statistical analysis, the experiment must be performed with more samples. In this way, the T-Student hypothesis test, for sample size *n* = 2^31^, shows the significance (or not) of the differences in the results found. This test was performed with the aim of validating the hypothesis that there are no significant differences between the results found in the two methods. As previously discussed, it was possible to validate this hypothesis for the two samples with a significance level of 95%.

For the three-dimensional analysis, average roughness results ($R_a$) were obtained in the 2D linear profiles and compared with the three-dimensional results of $R_a$ for the surface of the entire rock sample. In a general analysis, it is possible to observe that, for two rock samples, the linear results presented values of $R_a$ higher than those in all quadtrees present on the surfaces of the rock samples. In addition, the quadtrees with the highest $R_a$ do not coincide with the main diagonal of the samples (where the 2D linear results of $R_a$ were obtained—Figs. 8 and 9). These factors highlight the hypothesis that 3D results are essential in geomechanical studies due to the anisotropic nature of the rock surface.

Through the results, obtaining the coefficient $R_a$ contributes significantly to the application of the methodology in geomechanical studies. Through the $R_a$, it is possible to obtain the average roughness across the entire surface of rocks or rocky outcrops. The main advance of this method to the published and described studies is the ease of increasing (or decreasing) the accuracy of the results according to the user interest, changing the division/quantity of quadtrees analyzed.

## Conclusion

The roughness is essential in geomechanics study due to the influence on mechanical resistance, hydraulic flow, and fluid percolation^1^. Thus, the roughness of the rock surface is fundamental for research that seeks to evaluate and quantify the flow of fluids in geological formations and for the identification, classification, and study of natural fractures in wells as a means of storage of crude oil^6,8,10,32^.

This research had a study hypothesis that the data obtained by the three-dimensional roughness measurements are important in geomechanics due to the non-regularity of the rock surface. Through this hypothesis, the objective was to adapt and apply a method of hierarchical division in three-dimensional digital representations of rocks to determine the roughness coefficient, comparing the results through two-dimensional readings performed through a conventional method.

Through our methodology, it was possible to confirm the hypothesis and emphasize the importance of carrying out a three-dimensional analysis of the rock surfaces in their entirety. The results show very distinct roughness values ($R_a$) along its surface. Furthermore, the results obtained through the two-dimensional reading were different from those in the three-dimensional analysis. Thus, we confirm that this difference is significant in geomechanical studies. The use of hierarchically organized $R_a$ is significant for the results of the 3D surface roughness of a rock sample, as this method allows altering the level of details according to the user’s interest. The methodology also provides locally and globally analyzed roughness results showing that the average surface roughness of rock samples is variable according to the analyzed scale.

This research has the potential to advance to use of the 3D roughness results from this method as a variable in fluid flow and formation of fractures calculations. These results will be associated with porosity, permeability, and rock wear data. Furthermore, the results from valley areas can be applied to the direction of fluid flow within the rock through the resulting vector. Another approach for future research is to use more rock samples to promote a more statistically reliable analysis and use direct acquisition methods such as a 3D scanner to the accuracy of 3D surface acquisition data.

## Data Availability

The datasets that were generated and/or analysed during the current study are freely available from the corresponding author on a request.
